# Extracellular vesicles produced by human-induced pluripotent stem cell-derived endothelial cells can prevent arterial stenosis in mice via autophagy regulation

**DOI:** 10.3389/fcvm.2022.922790

**Published:** 2022-10-17

**Authors:** Yecheng He, Quanfu Li, Feng Feng, Rupan Gao, Huadong Li, Yuxin Chu, Shaobo Li, Yin Wang, Ruoying Mao, Zhongzhong Ji, Yutao Hua, Jun Shen, Ziao Wang, Meng Zhao, Qing Yao

**Affiliations:** ^1^Department of Clinical Medicine, Suzhou Vocational Health College, Suzhou, Jiangsu, China; ^2^Hubei Key Laboratory of Diabetes and Angiopathy, Medicine Research Institute, Xianning Medical College, Hubei University of Science and Technology, Xianning, Hubei, China; ^3^Department of Anesthesiology, Shanghai Pulmonary Hospital, Tongji University School of Medicine, Shanghai, China; ^4^Institute of Physical Education, Inner Mongolia Normal University, Hohhot, Inner Mongolia, China; ^5^Department of Hematology, Zhongshan Hospital, Shanghai Medical College of Fudan University, Shanghai, China; ^6^Department of Cardiovascular Surgery, Union Hospital, Tongji Medical College, Huazhong University of Science and Technology, Wuhan, Hubei, China; ^7^Department of Medicine, University of Alabama at Birmingham, Birmingham, AL, United States; ^8^School of Life Sciences, Westlake University, Hangzhou, Zhejiang, China; ^9^Department of Pharmacy, Suzhou Vocational Health College, Suzhou, Jiangsu, China; ^10^School of Life Sciences, Xiamen University, Xiamen, Fujian, China

**Keywords:** arterial restenosis, hiPSC-EC, autophagy, extracellular vesicles, miR-126

## Abstract

Intravascular transplantation of human-induced pluripotent stem cells (hiPSCs) demonstrated a significant therapeutic effect in the treatment of restenosis by the paracrine function of extracellular vesicles (EVs). However, the risk of tumorigenicity and poor cell survival limits its clinical applications. In this study, we for the first time applied a highly efficient and robust three-dimensional (3D) protocol for hiPSC differentiation into endothelial cells (ECs) with subsequent isolation of EVs from the derived hiPSC-EC (ECs differentiated from hiPSCs), and validated their therapeutic effect in intimal hyperplasia (IH) models. We found that intravenously (iv) injected EVs could accumulate on the carotid artery endothelium and significantly alleviate the intimal thickening induced by the carotid artery ligation. To elucidate the mechanism of this endothelial protection, we performed miRNA expression profiling and found out that among the most conserved endothelial miRNAs, miR-126 was the most abundant in hiPSC-EC-produced EVs (hiPSC-EC-EV). MiR-126 depletion from hiPSC-EC-EV can hinder its protective effect on human umbilical vein endothelial cells (HUVECs) in an inflammatory process. A variety of functional *in vitro* studies revealed that miR-126 was able to prevent endothelial apoptosis after inflammatory stimulation, as well as promote EC migration and tube formation through autophagy upregulation. The latter was supported by *in vivo* studies demonstrating that treatment with hiPSC-EC-EV can upregulate autophagy in mouse carotid artery ECs, thereby preventing IH and modulating vascular homeostasis via remodeling of the vascular intima. Our findings suggest a regulatory mechanism for the therapeutic effect on arterial restenosis by autophagy regulation, and provide a potential strategy for clinical treatment of the disease.

## Introduction

Arterial restenosis represents a major complication in vascular reconstructive procedures, such as balloon angioplasty, bypass grafting, stent angioplasty, and endarterectomy. Stimuli, such as an altered blood flow, mechanical load, and vascular endothelial injury in the postoperative period, followed by recruitment and infiltration of inflammatory cells into the vascular walls and subsequent secretion of cytokines and growth factors lead to the vascular smooth muscle cell (VSMC) proliferation and migration, and result in IH, which further progresses into vascular restenosis (1–4). Therefore, the exploration of effective strategies for IH intervention is necessary to prevent postoperative restenosis.

Recently, stem cell therapy showed great promise for curing restenosis. HiPSCs are generated by reprogramming somatic cells and represent a source of autologous pluripotent stem cells. Since the discovery of induced pluripotent stem cells (iPSCs) in 2006 (5), transplantation of iPSCs and differentiated derivatives thereof emerged as a promising direction in regenerative therapy, owing to the high pluripotency of iPSCs, low immune rejection response to them, and the lack of ethical restrictions associated with their use (5, 6). However, due to some disadvantages of stem cell therapy, such as low cell survival rate, genomic instability, and potential tumorigenicity, as well as a limited targeting efficiency (7, 8), advanced preclinical studies have recognized that paracrine factors generated by transplanted iPSCs, rather than the cells *per se*, confer the major beneficial effects of regeneration on the injured tissues (9, 10).

Among these paracrine molecules, EVs exhibit unique functions and hold great potential in disease diagnosis and therapy. EVs are extracellular membrane vesicles secreted by various cell types (11). EVs contain miRNAs and other components capable of regulating cell proliferation or apoptosis in vascular endothelium and promoting remodeling of the vascular intima. EVs enable cells to modulate molecular mechanisms remotely, i.e., exert molecular effects beyond the intracellular compartments of the effector cell (12–14).

Extracellular vesicles used for vascular healing in previous studies were primarily derived from mesenchymal stem cells (MSCs) or other types of stem cells (15, 16). The impairment of the EC function was believed to be a major regulatory factor in the restenosis mechanism. Although ECs are also likely to generate EVs with therapeutic effects, they are rarely used as an EV source due to the lack of sustainable expandability and quality cell lines. In this study, we employed a 3D differentiation protocol, which can efficiently generate functional ECs from hiPSCs (17). We then isolated EVs from hiPSC-EC and injected them into mice IH models to evaluate their therapeutic effect and identify the disease treatment mechanisms. Compared with MSCs, the major advantages of hiPSCs include that they can be mass-produced and individualized for treatment. Meanwhile, using a microfluidic chip to simulate the circulatory system *in vivo*, we have also demonstrated that hiPSC-EC-EV have better cellular adhesion capacity and therapeutic effect than HUVEC-EV (Supplementary Figure 1). Since EC differentiation and hiPSC-EC-EV production using our protocol are remarkably more efficient and robust than those achieved by conventional methods, our results offer a potential strategy for the clinical treatment of vascular restenosis.

## Materials and methods

### Establishing an animal model for intimal hyperplasia

The C57 mice (cleaning grade) weighing 25–28 g were selected to prepare the arterial injury model. All mice were kept under specific pathogen-free conditions in strict compliance with the facility standards approved by the China Laboratory Animal Management Accreditation Association. The mice were anesthetized by intraperitoneal injection of sodium pentobarbital (40 mg/kg) and placed on the operating table. The animals’ neck skin was sterilized with iodine wine and alcohol and then cut off. The subcutaneous tissue and muscle were separated successively, the left common carotid arteries were exposed, and the bifurcation of the internal/external branches was ligated completely with a 5-0 silk suture. At last, the subcutaneous tissue and skin were sutured layer by layer. All mice were maintained at the Hubei University of Science and Technology.

### Cell culture of human-induced pluripotent stem cells

Human iPSCs were obtained from Dr. Pan’s laboratory at the Tenth People’s Hospital affiliated with Tongji University, where they were generated by reprogramming the human dermal fibroblasts from a 40-year-old volunteer with informed consent. All cell lines were propagated in feeder-free growth conditions. Briefly, cells frozen in a cryopreservation tube were removed from liquid nitrogen and transferred to a 37°C water bath for thawing. Then they were mixed with 2 ml of resurviving medium (mTeSRI + 10 μM Y-276362) (StemCell Technologies, Vancouver, BC, Canada) and centrifuged at 300 g for 5 min. The supernatant was discarded, and the cells were mixed with 2 ml of resurviving medium, transferred onto a Matrigel (Corning)-coated six-well plate, and placed in the incubator for 24 h. On the next day, the medium was replaced with 2 ml of fresh mTeSRI without Y-27632.

### Endothelial cell differentiation

**FIGURE 1:**
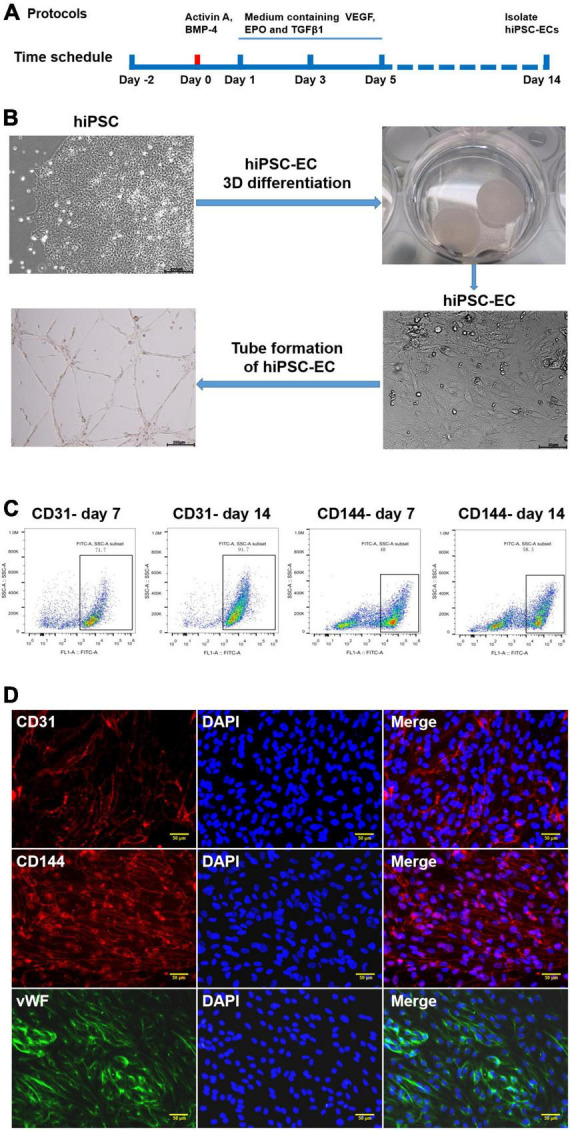
Generation and characterization of human-induced pluripotent stem cell-endothelial cells (hiPSC-ECs). **(A)** Schematic representation of the protocol for differentiation of hiPSCs to ECs; **(B)** Differentiation of hiPSC-EC through a 3D protocol; **(C)** Flow cytometry assay for the detection of CD31 and CD144 expression post-hiPSC-EC differentiation; **(D)** Identification of hiPSC-EC by immunofluorescent staining.

The EC differentiation was performed as described previously (17), and the protocol is schematically outlined in Figure 1A. Three days before initiating the differentiation procedure, hiPSCs were dissociated into single cells and seeded into a 0.5 ml fibrin scaffold patch in a 24-well plate for 48 h. On day 0, the cell-containing fibrin scaffold was cultured in an EBM2 medium (Lonza) supplemented with B27 minus insulin, activin-A, and bone morphogenetic protein 4 (BMP-4) for 24 h. On day 1, the medium was replaced with an EBM2 medium supplemented with B27 minus insulin, vascular endothelial growth factor (VEGF), erythropoietin (EPO), and transforming growth factor β1 (TGFβ1) for another 4 days (the medium was refreshed on day 3). On day 5, the patch was treated with collagenase IV to release the differentiating cells, and the medium was replaced with EGM2-MV medium (Lonza) supplemented with B27, VEGF, and SB-431542. The medium was replaced every 2 days thereafter. Fluorescence-activated cell sorting (FACS) was performed on day 14 to evaluate the differentiation efficiency by analyzing the CD31 and CD144 expression levels in the differentiated cells. The CD31^+^CD144^+^-sorted hiPSC-ECs were cultured in an EGM2-MV medium supplemented with B27, VEGF, and SB-431542.

### Flow cytometry

Flow cytometry was performed as previously described (17). In brief, differentiated hiPSC-ECs were gently dissociated with trypsin (0.05%) and neutralized with the culture medium. After centrifugation and discarding of supernatant, the cells were incubated with FACS buffer (2% FBS in PBS) containing primary PE-conjugated anti-CD31 antibodies or FITC-conjugated anti-CD144 antibodies for 30 min at 4°C. Isotype antibodies were used as control. Then the cells were washed with FACS buffer, resuspended in 0.5 ml of FACS buffer, and analyzed with a FACS instrument (Sony MA900 Cell Sorter).

### Extraction and identification of human-induced pluripotent stem cells-endothelial cell-extracellular vesicle

The HiPSC-ECs were cultured to 70% confluency, and the supernatant was collected and centrifuged at 2,000 g for 10 min at 4°C to remove the residual cells and debris followed by another centrifugation at 100,000 g for 1 h at 4°C. Afterward, the EV pellets were resuspended in 50–100 μl of phosphate-buffered saline (PBS). EVs were visualized with a transmission electron microscopy (FEI Tecnai G2 Spirit, ThermoFisher Scientific, Waltham, MA, USA) directly or an inverted fluorescence microscope (Olympus FV3000). The diameter distribution of EVs was determined by NanoSight (Malvern Instruments Inc., Malvern, UK). Some specific protein markers, such as CD9, CD63, and TSG101, were identified by Western blotting analysis.

### Transmission electron microscopy

The extracted EVs were first fixed with 2.5% glutaraldehyde and then dropped onto the copper mesh, followed by staining with a 12% phosphor-tungstic acid aqueous solution (pH = 6.5). Afterward, the samples were visualized under the electron microscope, and the target pictures were taken.

### Lipophilic dye (PKH) labeling of extracellular vesicles

The resuspended EVs were stained with PKH26 (or PKH67) (Red Fluorescent Cell linker for General Cell Membrane) (Sigma-Aldrich, St. Louis, MO, USA). PKH26 dye was added to 100 μl of diluent C to a final concentration of 8 μM. Then 8 μg of EVs suspended in 20 μl of DPBS was added to 80 μl of diluent C, mixed with the PKH26 solution, and incubated at 37°C for 15 min. Afterward, 10% EV-depleted fetal bovine serum (FBS) diluted in DMEM was added to bind with excess dye, followed by diluting to 1 ml with PBS and centrifuging at 100,000 × g for 1 h at 4°C. The pellet was resuspended in 40 μl of PBS.

### Western blotting analysis

Western blotting was performed as described previously (18). In brief, total protein was extracted from the EVs or cells, and the concentration was determined by a BCA Protein Assay Kit (Beijing Solarbio Science and Technology Co., Ltd., Beijing, China). Total protein obtained from each sample in a lysis buffer was loaded on each lane and run on an SDS-PAGE for 1 h at 100 V, and then transferred onto a polyvinylidene fluoride membrane. The membrane was blocked with 5% milk for 1 h and then incubated with a primary antibody (Table 1) overnight at 4°C. HRP Affini-Pure Goat anti-Rabbit IgG (cat. # ab97051, Abcam) was used as a secondary antibody. Finally, the blots were visualized by enzyme-linked chemiluminescence assay (Beyotime, Shanghai, China) and analyzed by Image J software.

**TABLE 1:** Antibodies summary.

Antibody	Brand	Cat. no.
anti CD9	abcam	ab236630
anti CD63	abcam	ab134045
anti CD31	abcam	ab182981
anti CD144	abcam	ab33168
anti vWF	abcam	ab6994
anti Alix	abcam	ab275377
anti TSG101	abcam	ab133586
anti Bcl-2	abcam	ab182858
anti Ki67	abcam	ab16667
anti α-SMA	abcam	ab7817
anti P-mTOR (Ser2448)	CST	5536T
anti mTOR	CST	2983
anti Bax	CST	2772S
anti β-actin	CST	4970
anti LC3B	CST	2775S
anti P62	CST	39749S

### miRNA transfection and depletion of miR-126 on endothelial cell

In the miRNA transfection experiment, HUVECs were seeded in six-well plates first and grown to the confluency of about 70%. Then the cells were transfected with miR-126 mimic (Genepharma, Shanghai, China) and a control mimic (NC) by using Lipofectamine 2000 (Invitrogen, Carlsbad, CA, USA), according to the manufacturer’s recommendations. The supernatant was replaced with a complete culture medium after 5 h, and Western blotting was performed to assess the transfection effect after 48 h.

In the miR-126 depletion experiment, the hsa-miR-126/inhibitor and inhibitor negative control oligonucleotides were purchased from GenePharma. Cells in the logarithmic growth phase were trypsinized, counted, and seeded in six-well plates to ensure 50% cell confluence on the next day for transfection. Transfection of cells with oligonucleotides was performed using Lipofectamine™ 2000 Reagent in line with the manufacturer’s instructions (Invitrogen, Carlsbad, CA, USA) at a final concentration of 100 nM. After transfection with hsa-miR-126/inhibitor in hiPSC-EC cells, EVs were extracted from hiPSC-EC cells.

### Scratch test

The HUVECs were seeded in six-well plates and transfected with either a non-coding (NC) control miRNA or miR-126 mimic (300 nM) and cultured to 80–90% confluency. Then a sterile pipette tip was used to inflict a linear scratch injury in the center of the cell monolayer. Then the cells were cultured in a normal medium for another 48 h. The wound was monitored by a phase-contrast microscope (Olympus IX71), and the cell closure was determined by measurements with Image J software.

### Tube formation

The HUVECs were treated with either hiPSC-EC-EV or different miRNA mimics for 48 h before performing tube formation assays. Culture plates (24-well) were pre-coated with Matrigel (BD Pharmingen, Piscataway, NJ, USA), and then 5 × 10^4^/well HUVECs were seeded and cultured for 12 h. The proliferation and migration properties of the HUVECs were determined by quantification of the total tubule length in five random view fields per well.

### Hematoxylin and eosin staining and immunofluorescent staining

The HiPSC-ECs in chamber slides were treated with miR-126 mimic or control mimic for 48 h. Then the cells were washed with PBS and fixed with 4% paraformaldehyde (PFA) at room temperature for 15 min, and 0.2% Triton X-100 was added to permeabilize the membrane for 10 min. Then cells were blocked with 5% goat serum and incubated with primary antibodies (Table 1) overnight at 4°C. On the next day, the cells were incubated with secondary antibodies for 1 h and stained with DAPI (Sigma-Aldrich, St. Louis, MO, USA) for 5 min at room temperature. For mouse artery tissue analysis, excised arteries were fixed in 4% PFA for 1 h, dehydrated in 30% sucrose solution, embedded in OCT, and sliced into 5 μm sections. The sections were then stained with hematoxylin and eosin (H&E). Immunofluorescence analysis was performed as described above. The cells or the tissue sections were visualized by a confocal fluorescence microscope (Olympus FV3000).

### Fabrication of the microfluidic chip

The microfluidic chip consists of a PDMS layer and a glass substrate. First, the PDMS layer was fabricated by replica molding on a master by spin-coating SU8-2035 negative photoresist (Microchem Corp., Newton, MA, USA) onto a glass wafer and patterned by photolithography. After the master is prepared, the Sylgard 184 PDMS base and curing agent (Sylgard Silicone Elastomer 184, Dow Corning Corp., Midland, MI, USA) are thoroughly mixed, degassed under vacuum, and poured onto the master. The PDMS layer was incubated in an oven at 80°C for 1 h and peeled off from the master. The inlet and outlet holes are made by punching the PDMS with a razor-sharp punch. After 60 s of oxygen plasma treatment, this piece of PDMS was irreversibly bound to the glass substrate.

### Flow experiment in microfluidic chip

The microfluidic channels were pre-coated with 10 μg/mL of MaxGel human extracellular matrix extracts (Sigma-Aldrich, St. Louis, MO, USA) before seeding of cells. After coating, HUVECs (Lonza) were cultured inside the microfluidic channels at a density of 5 × 10^6^ cells/ml to produce a confluent layer. The fresh culture medium was replaced every 6–8 h. Until cell confluence, HUVEC monolayers were inflamed with TNF-α for 12 h. Then, the medium containing 1 μg/ml of PKH67-labeled HUVEC-EV or hiPSC-EC-EV was perfused through the channels for 6 h. The perfusion was performed with a programmable syringe pump connected with a microfluidic device which provides precise control of the flow rate at 20 μl/min. After 6 h, a blank medium without PKH67-labeled EVs was used to perfuse for 0.5 h. The uptake of fluorescently labeled EVs could be visualized under fluorescence microscopy.

### Ultrasonic vocalization recording

Mice were anesthetized under 2.5% isoflurane and then placed on a special console (animal handling and physical monitoring platform), and isoflurane concentration was adjusted to the maintenance level of 1.0–1.5%. Mouse ultrasound imaging was performed with the Vevo LAZR photoacoustic micro-ultrasound imaging system (FujiFilm, VisualSonics Inc., Tokyo, Japan). Once the carotid artery was clearly displayed, the probe was adjusted so that the ultrasonic beam was perpendicular to the anterior and posterior walls of the left common carotid artery, and the intima-media of the artery’s anterior and posterior walls was displayed. High-resolution anatomic carotid allograft images were generated with the MS550D transducer at 40 MHz.

### Statistical analysis

The IBM SPSS 22 software version was used for statistical analysis. An unpaired *t*-test and one-way ANOVA were used to calculate statistical significance. The results are expressed as a mean ± SD with *P* < 0.05 indicating the statistical significance of the differences.

## Results

### Generation and identification of human-induced pluripotent stem cell-endothelial cells

Human iPSCs represent an unlimited source of cells for replacing damaged tissues. They can be successfully differentiated into different subtypes of vascular cells, such as ECs (19, 20) and smooth muscle cells (SMCs) (21, 22). In this study, we used an efficient method for differentiating hiPSCs to CD31^+^ CD144^+^ ECs as a source of EVs (Figure 1A). HiPSCs were embedded in a biodegradable scaffold suspended in a mTeSR stem cell medium for 3 days before culturing in endothelial growth medium EBM2 supplemented with activin A and BMP-4, as well as some other growth factors following differentiation, which gave rise to endothelial-like cells, as demonstrated by a cobblestone morphology and the formation of a robust network of tubular structures upon seeding on Matrigel (Figure 1B). The efficiencies of hiPSC-EC differentiation were evaluated at day 7 and day 14 by performing flow cytometry analysis. On day 7, the expression of CD31 and CD144 in the differentiating ECs was 71.7 and 48%, respectively. On day 14, the EC differentiation efficiency increased to 91.7% for CD31 and 58.3% for CD144 (Figure 1C). Immunostaining assay further demonstrated that day 14 was the ideal time point for EC isolation when the endothelial-specific genes for CD31, CD144, and vWF proteins exhibited very high expression levels (Figure 1D).

### Characterization of human-induced pluripotent stem cell-endothelial cell-extracellular vesicle

**FIGURE 2:**
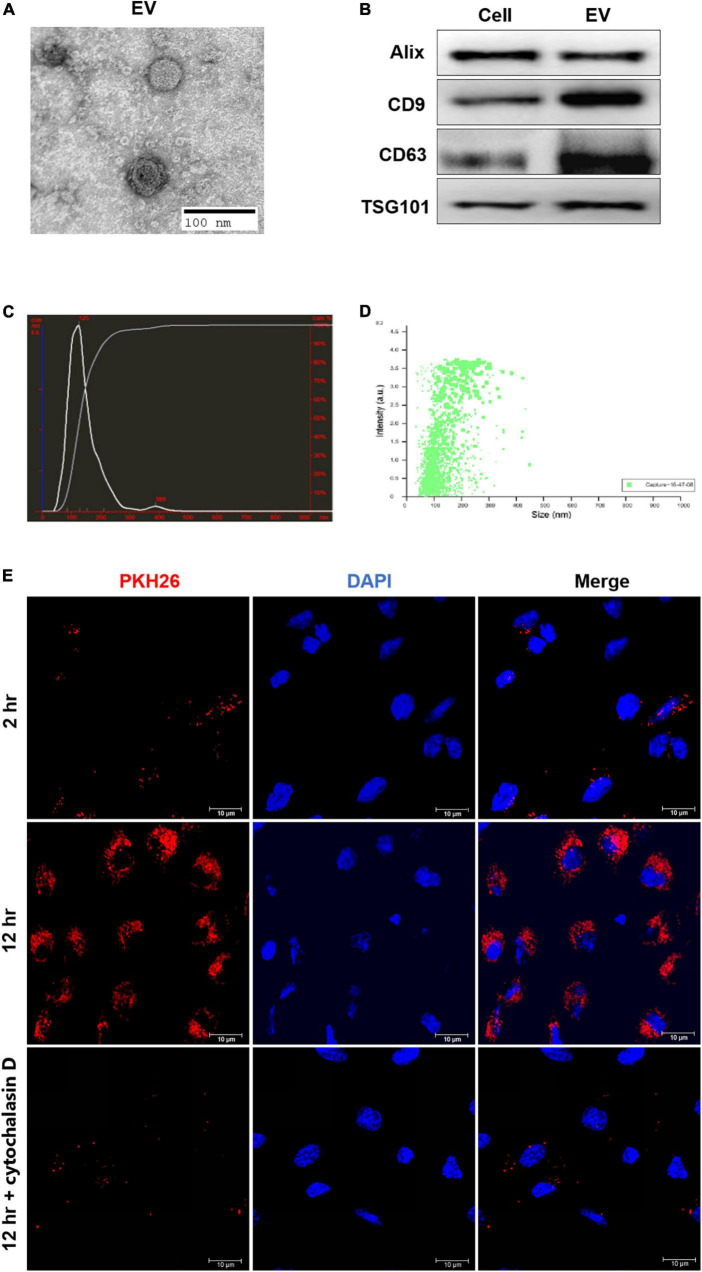
Isolation and characterization of human-induced pluripotent stem cell- endothelial cell-extracellular vesicle (hiPSC-EC-EV). **(A)** A representative micrograph of hiPSC-EC-EV visualized by transmission electron microscopy; **(B)** Relative expression levels of Alix, CD9, CD63, and TSG101 proteins in hiPSC-ECs and hiPSC-EC-EV, as revealed by Western blotting; **(C,D)** The hiPSC-EC-EV particle size distribution as measured and analyzed by NanoSight; **(E)** Representative confocal microscopy images of HUVECs exposed for either 2 or 12 h to PKH26-labeled hiPSC-EC-EVs with or without cytochalasin D treatment. The nuclei were counterstained with DAPI.

To validate the harvested hiPSC-EC-EV, the morphology of EVs was first analyzed by TEM. The EVs exhibited a roughly cup-shaped morphology with a diameter of 80–130 nm (Figure 2A). The mean diameter was around 125 nm, as determined by NanoSight (Figures 2C,D). Aside from the EV shape and size validation, Western blotting revealed the expression of some EV-associated proteins, such as Alix, CD63, and TSG101 (Figure 2B), so the EVs were validated as genuine. Furthermore, we observed PKH26 fluorescence in HUVECs at 2 h after incubation with EVs, while the fluorescence intensity became much higher after 12 h (Figure 2E). To determine whether the EVs could be taken up by cells or just adhered to the cell surface, we incubated HUVECs with PKH26-labeled hiPSC-EC-EV in the presence of endocytic inhibitor cytochalasin D for 12 h. We could see that the increase of PKH26 fluorescence could be interrupted by endocytic inhibitor cytochalasin D (Figure 2E), which demonstrated that over time of incubation, more EVs will be internalized into the cells.

### Luminal stenosis in injured carotid arteries is alleviated by treatment with human-induced pluripotent stem cell-endothelial cell-extracellular vesicle

**FIGURE 3:**
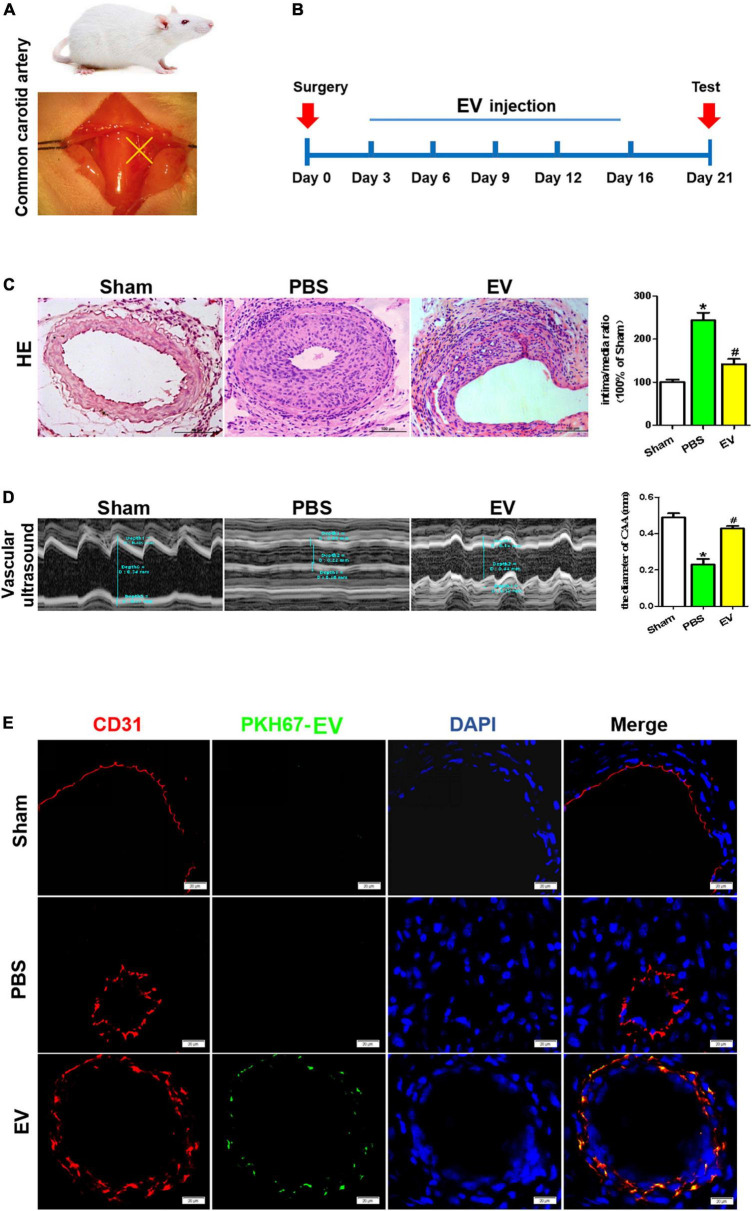
Arterial stenosis of carotid artery is reduced by exposure to human-induced pluripotent stem cell- endothelial cell-extracellular vesicle (hiPSC-EC-EV). **(A)** Schematic illustration of the carotid artery ligation procedure in the mouse model. **(B)** Time chart for the *in vivo* treatment of the vascular tissue with hiPSC-EC-EV and its collection for testing. **(C)** Analysis of the neointimal hyperplasia formation in the hiPSC-EC-EV-treated vs untreated mice 21 days after surgery by HE staining, and quantification of the intima-to-media ratio in the blood vessels. Scale bar = 100 μm. **(D)** Representative ultrasound images of the lumen size in the carotid arteries 21 days after the surgery and statistical analysis of the differences. **(E)** Representative images of ECs immunofluorescently stained for CD31 and PKH67 in the carotid arteries 21 days after the surgery. Data represent mean ± SD from three independent experiments (*n* = 3). Differences between linked groups were evaluated by a two-tailed Student’s *t*-test. *p < 0.05 vs. Sham, ^#^*p* < 0.05 vs. PBS.

The schematic diagrams illustrating the carotid artery ligation and the process for the hiPSC-EC-EV treatment are depicted in Figures 3A,B, respectively. Each group contained 7–9 mice. To evaluate the effect of hiPSC-EC-EV on luminal stenosis, we injected the EVs into the tail vein of mice (10 μg of EVs per injection and injected every 3 days), and the diameter of the carotid arteries in each mouse was analyzed 21 days after the surgery and EV treatment by HE staining and an ultrasound examination (Figures 3C,D). Compared with the sham group, the artery ligation induced a substantial increase in the neointimal layer’s thickness and the neointima-to-media ratio, although the EV treatment group also showed a higher index but obviously moderate (Figure 3C). Meanwhile, ultrasound examination also showed that the luminal diameter in the PBS group was significantly decreased and obviously recovered in the EV treatment group (Figure 3D). In addition, to further verify if the injected hiPSC-EC-EVs (green fluorescent PKH67-labeled) were taken up by the ECs located at the inner layer of the ligated carotid artery, PKH67 fluorescence was examined in CD31^+^ ECs. The immunostaining results showed that PKH67 fluorescence could only be found in the hiPSC-EC-EV treatment group and revealed an uptake of the injected hiPSC-EC-EV by the EC of the carotid artery (Figure 3E and Supplementary Figure 2). In conclusion, our results suggest a potential treatment effect of hiPSC-EC-EV on arterial stenosis induced by a vascular injury. In other major arterial vascular sites of the IH model, we did not detect the signals of EV (Supplementary Figure 2).

### Human-induced pluripotent stem cell-endothelial cell-extracellular vesicle could alleviate arterial stenosis by repressing endothelial cell apoptosis

**FIGURE 4:**
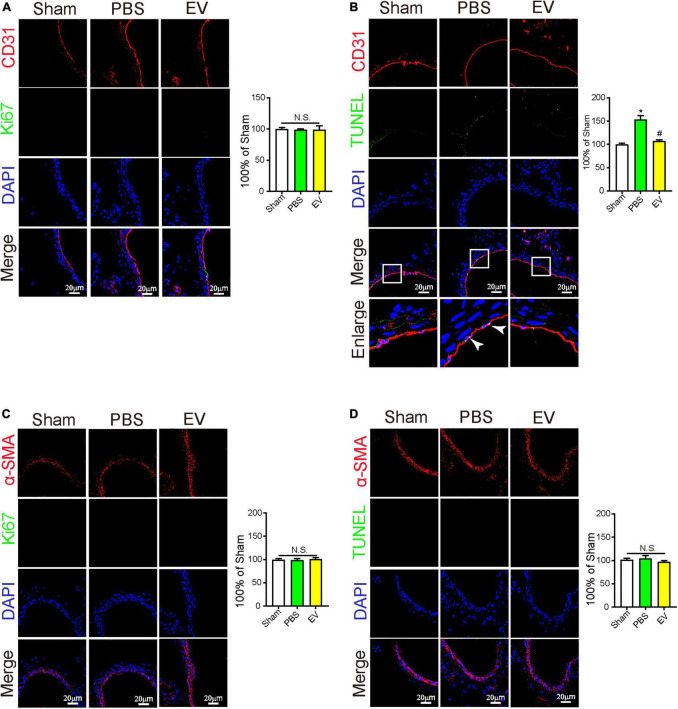
The protective effect of human-induced pluripotent stem cell- endothelial cell-extracellular vesicle (hiPSC-EC-EV) on carotid artery through inhibition of EC apoptosis. **(A–D)** Representative immunostaining images stained for CD31 (Red) and Ki67 (Green) in panel **(A)**, stained for CD31 (Red) and TUNEL (Green) in panel **(B)**, stained for α-SMA (Red) and Ki67 (Green) in panel **(C)**, and stained for α-SMA (Red) and TUNEL (Green) in panel **(D)** in the carotid arteries 21 days after the surgery. Data represent mean ± SD from three independent experiments (*n* = 3). Differences between linked groups were evaluated by two-tailed Student’s *t*-test. *p < 0.05 vs. Sham; ^#^*p* < 0.05 vs. PBS.

The overproliferation of SMCs and apoptosis of ECs exert important roles in arterial stenosis (1, 2). From the mouse *in vivo* study, we found that the apoptosis of ECs in the carotid artery was downregulated in the hiPSC-EC-EV treatment group compared with the PBS group (Figure 4B), but the SMC and EC proliferation showed no difference between the groups (Figures 4A,C). At the same time, may be due to the fact that SMCs are located in the middle layer of the arterial lumen wall and receive less blood fluid pressure, there was also no significant difference in the apoptosis of SMCs between the groups (Figure 4D). These results indicated that hiPSC-EC-EV might alleviate the arterial stenosis after ligation by repressing EC apoptosis.

### The protection effect of human umbilical vein endothelial cells elicited by the human-induced pluripotent stem cell-endothelial cell-extracellular vesicle treatment requires activation of autophagy

**FIGURE 5:**
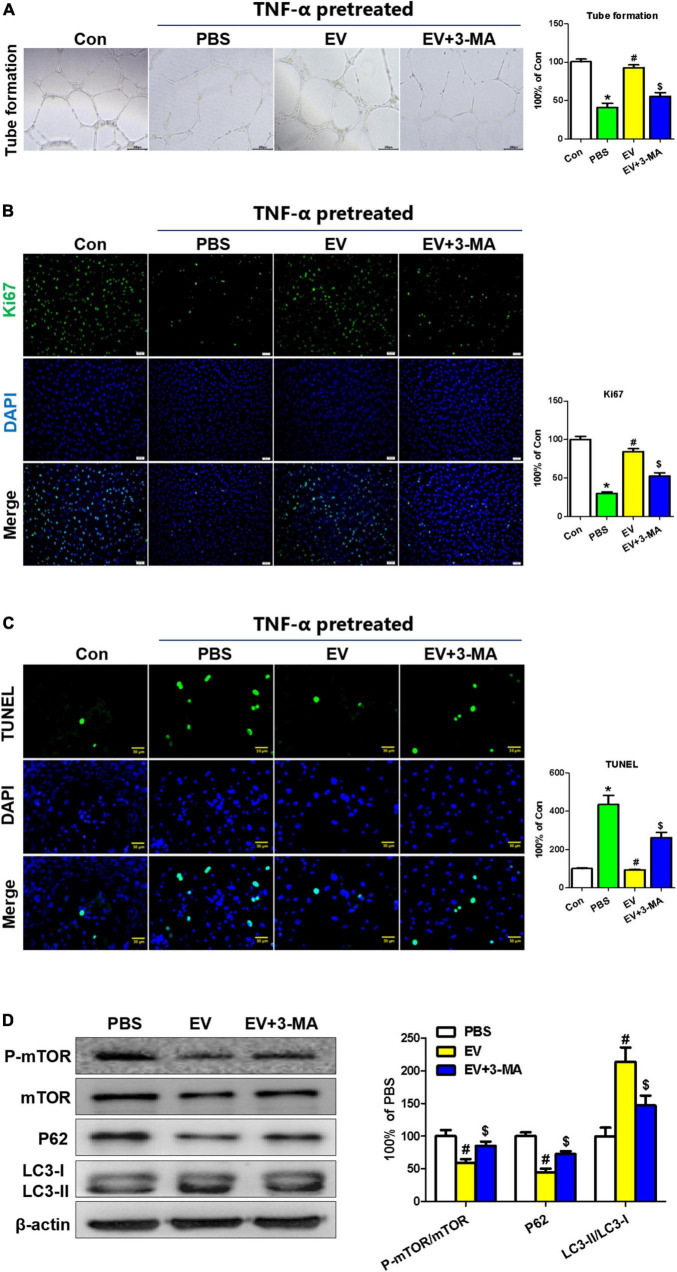
The protective effect of human-induced pluripotent stem cell- endothelial cell-extracellular vesicle (hiPSC-EC-EV) on TNF-α-damaged human umbilical vein endothelial cells (HUVECs). **(A)** Representative light microscopy images of the HUVECs in a tube formation assay. **(B)** Ki67 immunostaining and **(C)** TUNEL analysis in the HUVECs treated with or without EV and 3-MA in the presence of TNF-α. **(D)** Western blotting analysis of the autophagy-related protein expression in the HUVECs with different treatments. Data represent mean ± SD from three independent experiments (*n* = 3). Differences between linked groups were evaluated by a two-tailed Student’s *t*-test. *p < 0.05 vs. Con; ^#^*p* < 0.05 vs. PBS; ^$^*p* < 0.05 vs. EV.

To analyze the effect of the hiPSC-EC-EV treatment on the EC function, we detected the tubulation, proliferation, and apoptosis of HUVEC cells *in vitro*. Previous studies established that the inflammatory reaction plays an important role in neointimal hyperplasia (1). To simulate an inflammatory process *in vivo* and evaluate the protecting effect of hiPSC-EC-EV, relevant assays were performed in HUVECs in the presence of 10 ng/ml of TNF-α for 24 h (23). All the *in vitro* tests were repeated at least three times. The results showed that hiPSC-EC-EV could significantly increase the tube length on Matrigel and promoted the proliferation rate of HUVEC (Figures 5A,B). Furthermore, we found that the hiPSC-EC-EV treatment could alleviate cell apoptosis relative to the PBS group (Figure 5C), suggesting an important role of the hiPSC-EC-EV in endothelial protection. Previous studies have suggested a protective role of autophagy in cardiovascular injury (24). In our study, autophagy-related proteins were analyzed by Western blotting, and found that hiPSC-EC-EV treatment could improve the LC3-II/LC3-I ratio (Figure 5D). When autophagy inhibitor 3-MA (10 mM) was added to the cell culture medium following the hiPSC-EC-EV treatment, the enhanced tube formation and cell proliferation capability were interrupted, and the decreased cell apoptosis was also partially reversed, suggesting that the hiPSC-EC-EV-mediated protection is highly related to autophagy pathway, and an autophagy modulation could be used as a strategy to reduce arterial restenosis.

### miR-126 contained in human-induced pluripotent stem cell-endothelial cell-extracellular vesicle is a potential therapeutic target

**FIGURE 6:**
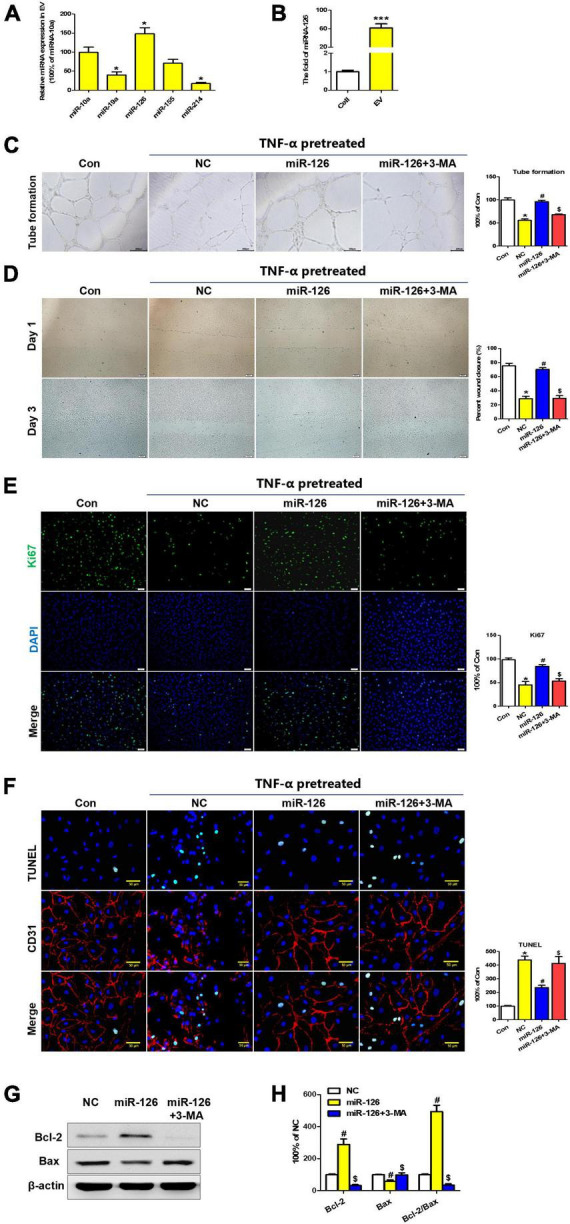
Extracellular vesicle (EV)-derived miR-126 promotes human umbilical vein endothelial cell (HUVEC) migration and survival via autophagy regulation. **(A)** The expression profiles of some common EC miRNAs; **(B)** The expression levels of miR-126 in hiPSC-EC and hiPSC-EC-EV; **(C,D)** Light microscopy (bright field) images of the HUVECs in a tube formation **(C)** and a scratch-wound **(D)** assay; **(E)** Ki67 staining (Green) of HUVECs treated with or without miR-126 and 3-MA in the presence of TNF-α; **(F)** TUNEL (Green) and CD31 (Red) staining of HUVECs treated with or without miR-126 and 3-MA in the presence of TNF-α; **(G)** Western blotting analysis of apoptosis-specific proteins in the treated HUVECs; **(H)** Densitometric quantification proteins from the Western blotting image pane in panel **(G)**; Data represent mean ± SD from three independent experiments (*n* = 3). Differences between linked groups were evaluated by a two-tailed Student’s *t*-test. *p < 0.05 vs. Con; ^#^*p* < 0.05 vs. NC; ^$^*p* < 0.05 vs. miR-126.

To elucidate the underlying mechanisms of EC protection by hiPSC-EC-EV, some miRNAs commonly expressed by ECs were identified by quantitative PCR (qPCR). We found that the expression level of miR-126 was significantly higher in hiPSC-EC-EV relative to other miRNAs (Figure 6A). On the other hand, miR-126 can be more highly enriched in hiPSC-EC-EVs than in hiPSC-ECs (Figure 6B). To demonstrate that the protective effects of EVs were at least partially mediated by miR-126, the hsa-miR-126/inhibitor oligonucleotides or NC oligonucleotides were transfected into hiPSC-ECs, and then the EVs in the supernatant were collected at 48 h post-transfection. As compared to NC-oligo EV, miR-126-depleted EV showed a weakened therapeutic effect as indicated by attenuated tubulation and proliferation, as well as enhanced apoptosis in inflammatory cytokine-stimulated HUVECs (Supplementary Figure 3).

Furthermore, a series of assays were conducted to verify if miR-126 has a protection effect for HUVEC in inflammatory reactions. As expected, tube formation and scratch-wound tests verified that transfection of HUVECs with miR-126 induced an enhancement of tubulation and migration in contrast to the negative control (NC) miRNA group. Importantly, these improvements could be abolished by treatment with autophagy inhibitor 3-MA (Figures 6C,D). On the other hand, miR-126 could also promote the proliferation and inhibit the apoptosis of HUVEC cells *in vitro* (Figures 6E,F). In addition, miR-126 was found to enhance the expression of anti-apoptosis protein Bcl-2 and suppress the expression of pro-apoptotic protein Bax, which could be reversed by autophagy inhibitor 3-MA (Figures 6G,H).

### miR-126 exerts therapeutic effect via autophagy regulation

**FIGURE 7:**
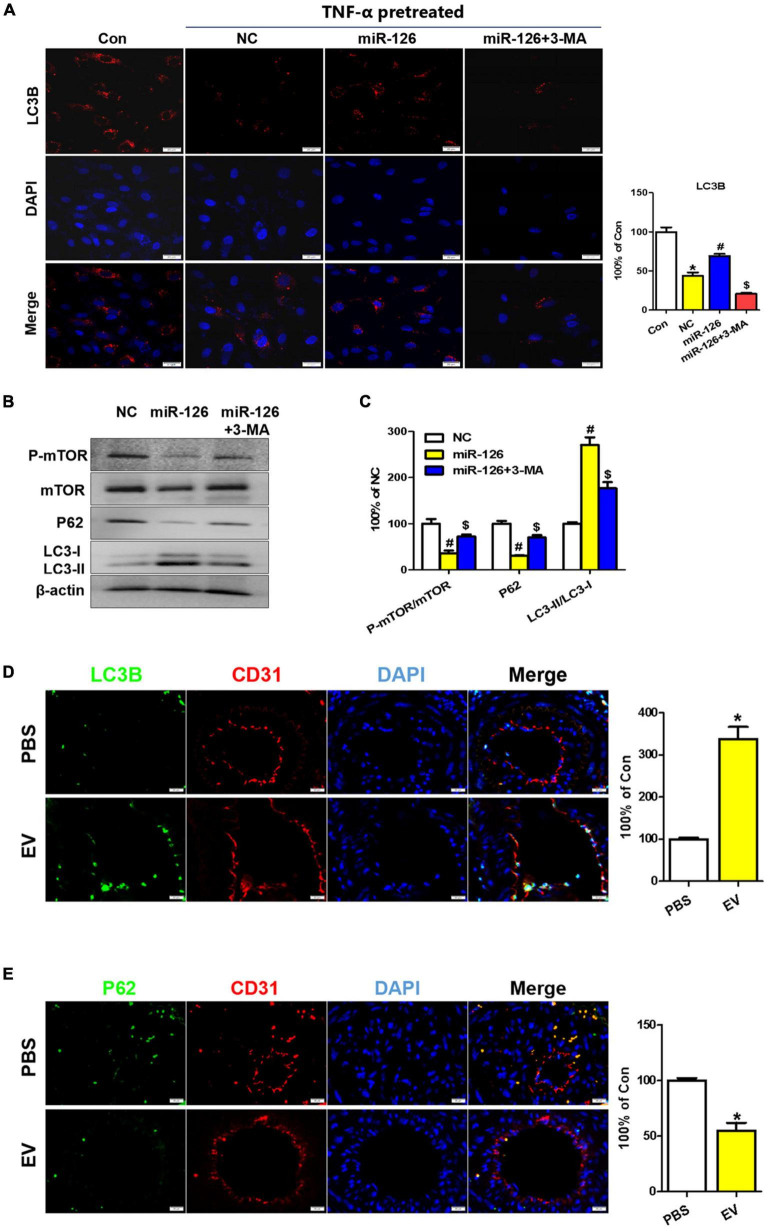
Upregulation of autophagy in EC of the carotid artery upon human-induced pluripotent stem cell- endothelial cell-extracellular vesicle (hiPSC-EC-EV) treatment. **(A)** LC3B staining in human umbilical vein endothelial cells (HUVECs) following treatment with or without EV and 3-MA in the presence of TNF-α and quantification of the LC3B-positive signals. **(B)** Western blotting analysis of the p-mTOR, mTOR, P62, and LC3 proteins in the treated and untreated HUVECs. **(C)** Densitometric quantification proteins from the Western blotting image pane in panel **(B)**. *p < 0.05 vs. Con; ^#^*p* < 0.05 vs. NC; ^$^*p* < 0.05 vs. miR-126. **(D,E)** Immunofluorescence staining of ECs for LC3B **(D)** and P62 **(E)** markers and their quantification (bar charts) in mouse carotid artery sections. Data represent mean ± SD from three independent experiments (*n* = 3). Differences between linked groups were evaluated by a two-tailed Student’s *t*-test. *p < 0.05 vs. PBS.

Some previous studies have reported the effect of miR-126 on autophagy regulation and the role of autophagy in cardiovascular diseases (25, 26). To further verify whether miR-126 exerted its protection effect via an autophagy regulation, Western blotting and immunofluorescence analyses were performed with HUVECs. The immunostaining showed that miR-126 significantly promoted the LC3B expression in HUVECs, while 3-MA suppressed this effect (Figure 7A). This was further confirmed by Western blotting, and miR-126 can remarkably increase the LC3-II/LC3-I ratio and suppress the expression of p-mTOR and p62 in HUVECs (Figures 7B,C). Taken together, these findings indicate that miR-126 enriched in EV can substantially promote the autophagy function of EC.

To assess the role of hiPSC-EC-EV-induced autophagy in facilitating the injury healing process in the carotid artery, we examined the endothelial autophagy level in murine carotid artery sections by immunofluorescence staining and found that the LC3B levels were consistently higher in the ECs of the hiPSC-EC-EV treatment group when compared with those of the PBS control group (Figure 7D and Supplementary Figure 4A), whereas the P62 expression was weaker (Figure 7E), demonstrating that autophagy was activated in EC by the hiPSC-EC-EV treatment. The mTOR is a master regulator of cellular metabolism, which also plays a crucial role in regulating autophagy. Activation of mTORC1 can lead to autophagy inhibition through the phosphorylation of multiple proteins (27). Our Western blotting analysis results demonstrated that hiPSC-EC-EV could reduce p-mTOR expression in HUVECs *in vitro* (Figure 7B). Here, we also found that a systemic administration of hiPSC-EC-EV into mice could inhibit the mTOR pathway in the EC of the carotid artery (Supplementary Figure 4C), implying its negative regulatory effect on autophagy. Taken together, these results demonstrate an increase in endothelial autophagy levels *in vivo* in response to the hiPSC-EC-EV treatment, which is possibly associated with the downregulation of the mTOR pathway.

## Discussion

**FIGURE 8:**
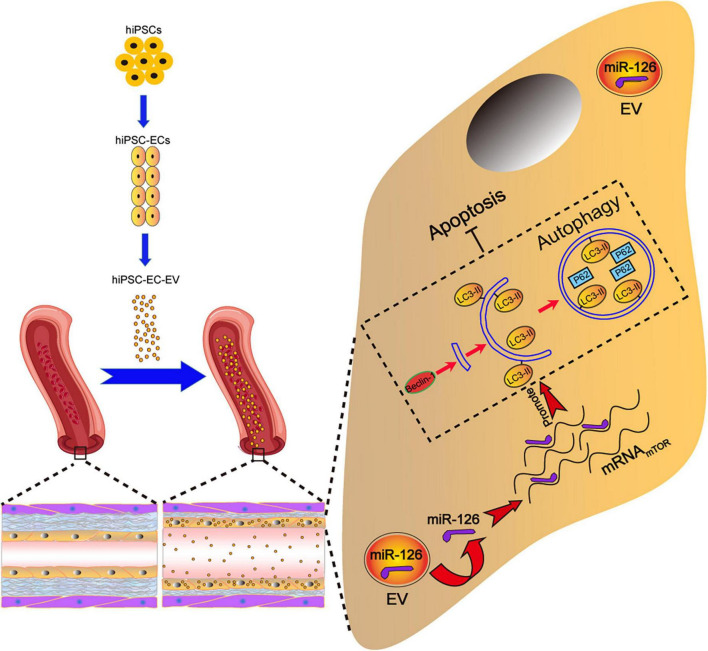
Schematic illustration of the tentative mechanism for alleviation of arterial restenosis by hiPSC-EC-EV treatment in mice via the miR-126-mediated autophagy regulation. Intravenously (iv)-injected hiPSC-EC-EV enriched in miR-126 could accumulate on the surface of the carotid artery endothelium, and suppress apoptosis in the ECs via autophagy upregulation.

In the present study, we investigated the protective effect of hiPSC-EC-EV on arterial restenosis conferred by the upregulation of the autophagy function in the arterial ECs. The results confirmed that hiPSC-EC-EV could alleviate ligation-induced arterial restenosis in a mouse model. To elucidate the underlying mechanism, we analyzed the functional role of EV-derived miRNAs and found that among them miR-126 exerted beneficial effects by enhancing proliferation, migration, and tube formation in the injured HUVECs, as well as by inhibiting HUVEC apoptosis via activation of the autophagy signaling pathway (Figure 8).

Shear stress, hypoxia, and arterial wall pressure inducing endothelial dysfunction are critical pathological factors observed in artery diseases, such as arterial restenosis (28). Current clinical strategies to reduce arterial restenosis include surgical treatment, drug therapy, cell therapy, etc. However, the therapeutic effects of such treatments are inefficient. Therefore, it is necessary to improve the current treatment approaches or find more effective new strategies.

In the current study, our team utilized an artery ligation technique to model endothelial dysfunction, and it was found that the ligation-treated arteries exhibited increased EC apoptosis. Our results are well consistent with the previous study which demonstrated that shear stress led to EC apoptosis and angiogenic dysfunction (29). MSC and some endothelial progenitor cell-based anti-stenotic therapies have been considered to be ideal interventions for the treatment of arterial restenosis (30–33). It has been demonstrated that MSCs have the ability to repair the shear stress-injured ECs by directly differentiating into functional ECs and secreting a number of angiogenic factors (34). However, cell therapy has many limitations of its own. For example, the low survival rate of transplanted cells and infusion toxicity caused by cell rejection seriously interfere with its therapeutic effect. The use of EVs secreted by stem cells allows avoiding those limitations. EVs are small enough to cross the tissue barrier, and yet allow a reduction in the infusion toxicity and unwanted tissue formation caused by stem cell therapy (35).

In contrast to MSC, hiPSCs have received wider attention with regard to their utility for transplantation therapy because of their self-renewing capability and pluripotency, which have revolutionized the cardiovascular therapy field. Studies have shown that transfer of hiPSC-derived EVs into blood vessels could increase microvessel density and blood perfusion in ischemic limbs *in vivo* (36). Considering the potential tumorigenicity of hiPSC, a better strategy is to differentiate hiPSC into ECs or EPCs and then isolate their EVs to perform the following treatment. It has also been reported that endothelial progenitor cells (EPCs)-derived EVs can significantly improve the endothelial and vascular repair of intervention-associated vascular injury (37). As compared to primary ECs or EPCs, one key advantage of hiPSC-EC is that it provides a sufficient and individualized resource for EV therapy.

It is known that the endothelial response to injury can be divided into two stages: initial, rapid response and a slower, phenotypic response. The initial or rapid response involves an EC apoptosis and dysfunction. The slower response involves impairment of angiogenesis and vascular remodeling in the artery (38). Our research demonstrated that hiPSC-EC-EV could promote wound healing and tube formation under inflammatory conditions, and significantly reduce EC apoptosis at an early stage, followed by reducing vascular remodeling and arterial restenosis at a later stage.

To validate the effects of hiPSC-EC-EV on shear stress-injured ECs, we co-cultured hiPSC-EC-EV with the HUVECs and found that the inflammation-induced EC dysfunction was significantly alleviated by the hiPSC-EC-EV treatment, as indicated by the upregulation of the EC proliferation, migration, and tube formation capability. It is well known that the migration and tube formation capabilities of ECs contribute to angiogenesis and the artery repair mechanism (39). In addition, EC apoptosis can lead to the dysfunction of vascular homeostasis and result in arterial restenosis (40).

A series of studies have demonstrated that MSC-EVs are rich in miRNAs, such as miR-126, miR-130a, miR-132, miR-210, and miR-378, and that miRNAs derived from hiPSC-EC-EV exert protective effects on the EC survival and artery function (41, 42). One of the most commonly found in EC miRNAs is miR-126, which exerts beneficial effects on angiogenesis, maintains vascular integrity, and provides atherosclerotic plaque stability (43, 44). In qPCR screening, we found that miR-126 was highly abundant in hiPSC-EC-EV. MiR-126-depleted EV showed a weakened therapeutic effect as indicated by attenuated tubulation and proliferation, as well as enhanced apoptosis in inflammatory cytokine-stimulated HUVECs. To further determine whether miR-126 was responsible for the protective effects observed from hiPSC-EC-EV, we performed a series of experiments to examine the role of miR-126 in regulating EC functions. Scratch and tube formation experiments demonstrated that ECs overexpressing miR-126 exhibited a better survival and an angiogenic potential, as compared to the control group. Additionally, a TUNEL assay-based analysis showed that the number of apoptotic cells in the miR-126 mimic group was significantly reduced, which was further evidenced by the decreased expression of apoptosis marker Bcl-2, indicating the protective role of miR-126 in ECs.

Previous reports have suggested that miR-126 can alleviate EC injury through modulating autophagy (45), which plays a key role in lipid metabolism and has been implicated in the pathogenesis of atherosclerosis (46). The present study showed that miR-126 mimic promotes the accumulation of autophagy marker LC3-II and reduces the expression of P62 protein in TNF-α-treated HUVECs, suggesting that miR-126 restores an impaired autophagic flux under inflammatory conditions. Furthermore, inhibition of the PI3K/Akt/mTOR signaling pathway was reported to upregulate cell autophagy (47). MiR-126 was reported to target PI3K (PI3Kp85b) and inhibit the PI3K/Akt/mTOR pathway, thereby rescuing an impaired autophagy flux (45). Interestingly, in this study, we found a suppression function of mTOR following miR-126 overexpression, which was in agreement with the above report.

Furthermore, overexpression of miR-126, following the inhibition of the autophagy flux by 3-MA, restored protection against the TNF-α-induced HUVEC injury, confirming the role of autophagic flux in the protective effect of miR-126. Immunofluorescence staining of mouse arterial tissues evidenced an elevated expression of autophagy marker LC3B, as well as reduced apoptotic signals in response to the hiPSC-EC-EV treatment of mouse arteries, which argues that hiPSC-EC-EV may alleviate, at least partially, the IH by increasing the autophagy levels in ECs.

In the aggregate, our study revealed that EC-secreted EVs, as natural carriers of miR-126, were capable of preventing an EC injury, highlighting an important regulatory role of autophagic flux in this process and offering a promising new therapeutic strategy for the treatment of arterial restenosis. Considering the feasibility of large-scale production of hiPSC-EC-EV in laboratory settings, our finding has an obvious practical significance for the development of low-cost clinical treatment of arterial restenosis.

## Data availability statement

The original contributions presented in the study are included in the article/Supplementary material, further inquiries can be directed to the corresponding authors.

## Ethics statement

The animal study was reviewed and approved by Ethics Committee of Hubei University of Science and Technology.

## Author contributions

QY, MZ, YHe, QL, and FF contributed to design the study. QY, YHe, QL, FF, HL, MZ, and RG analyzed data and prepared the manuscript. QY, YHe, MZ, QL, FF, RG, HL, YC, SL, YW, RM, ZJ, YHu, JS, and ZW contributed to do the experiments. QY and YHe contributed to fund it. All authors contributed to the article and approved the submitted version.

## Supplementary material

Supplementary Figure 1 Extracellular vesicle (EV) adhesion study on a microfluidic chip. **(A)** Schematic overview of the microfluidic chip. The microfabricated vessel wall device uses compartmentalized PDMS microchannels to form an organized co-culture of HUVECs, whereby physiological arterial strain and EV adhesion from the liquid flow can be recreated. **(B)** Top, brightfield images of microfluidic chips after seeding with HUVECs. Bottom, immunofluorescent images of the microfluidic chips adhering with PKH67-labeled EVs after 6-h flow; **(C)** Representative images of TUNEL staining for HUVEC in microfluidic chips after TNF-α treatment followed by 6-h-perfusion with medium containing hiPSC-EC-EV or HUVEC-EV.

Supplementary Figure 2 Extracellular vesicle (EV) localization in different arteries. Representative images for PKH67-labeled EV (Green) localization in the carotid artery, aorta, and femoral artery 21 days after the surgery.

Supplementary Figure 3 miR-126 plays a key role in the protective effect of hiPSC-EC-EV on HUVECs. **(A–C)** Representative images and quantification of **(A)** tube formation assay, **(B)** Ki67 immunofluorescence, and **(C)** TUNEL analysis of the HUVECs treated with normal EV or miR-126-depleted EV. Data represent mean ± SD from three independent experiments (*n* = 3). Differences between each group were evaluated by a two-tailed Student’s *t*-test. **p* < 0 05 vs. EV.

Supplementary Figure 4 The hiPSC-EC-EV treatment could upregulate the endothelial autophagy in carotid artery. **(A–C)** Representative immunofluorescence images for LC3B **(A)**, p-mTOR **(B)**, and mTOR **(C)** in the carotid arteries 21 days after the surgery.
