# Supplementary figures and images for: Extracellular vesicles produced by human-induced pluripotent stem cell-derived endothelial cells can prevent arterial stenosis in mice via autophagy regulation
Source: Front Cardiovasc Med. 2022 Oct 17;9:922790. doi: 10.3389/fcvm.2022.922790 (PMC9618599; doi:10.3389/fcvm.2022.922790)

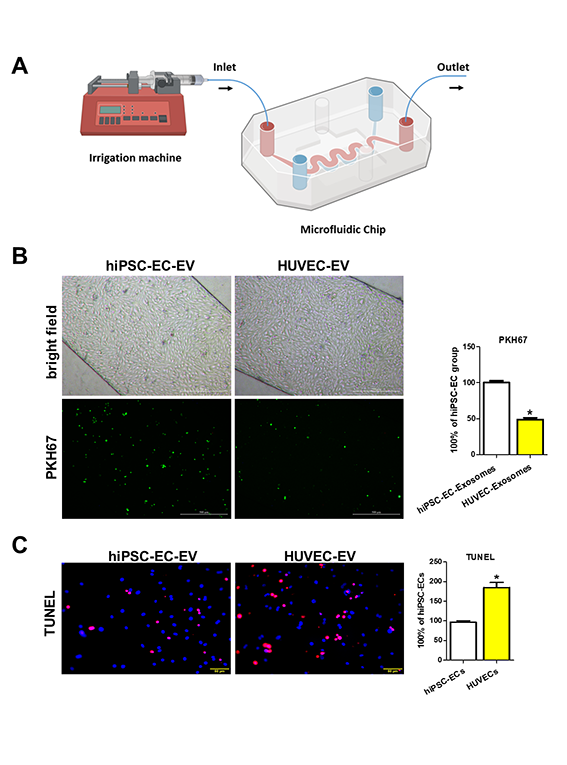

Supplement: Supplementary Figure 1 — Extracellular vesicle (EV) adhesion study on a microfluidic chip. (A) Schematic overview of the microfluidic chip. The microfabricated vessel wall device uses compartmentalized PDMS microchannels to form an organized co-culture of HUVECs, whereby physiological arterial strain and EV adhesion from the liquid flow can be recreated. (B) Top, brightfield images of microfluidic chips after seeding with HUVECs. Bottom, immunofluorescent images of the microfluidic chips adhering with PKH67-labeled EVs after 6-h flow; (C) Representative images of TUNEL staining for HUVEC in microfluidic chips after TNF-α treatment followed by 6-h-perfusion with medium containing hiPSC-EC-EV or HUVEC-EV. [file Image_1.tif]

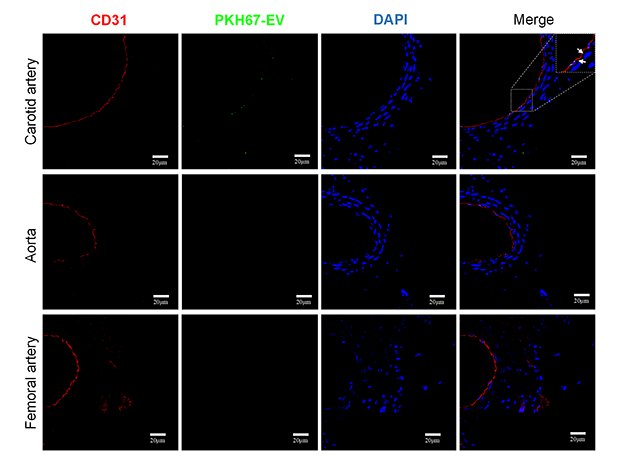

Supplement: Supplementary Figure 2 — Extracellular vesicle (EV) localization in different arteries. Representative images for PKH67-labeled EV (Green) localization in the carotid artery, aorta, and femoral artery 21 days after the surgery. [file Image_2.tif]

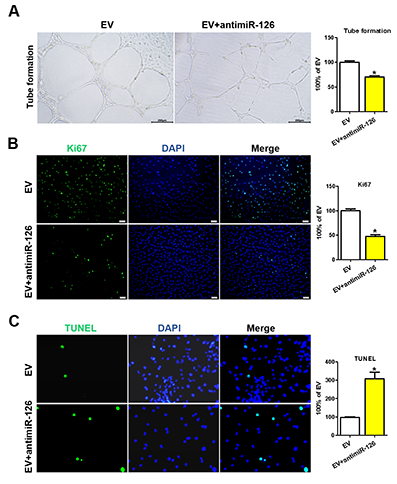

Supplement: Supplementary Figure 3 — miR-126 plays a key role in the protective effect of hiPSC-EC-EV on HUVECs. (A–C) Representative images and quantification of (A) tube formation assay, (B) Ki67 immunofluorescence, and (C) TUNEL analysis of the HUVECs treated with normal EV or miR-126-depleted EV. Data represent mean ± SD from three independent experiments (n = 3). Differences between each group were evaluated by a two-tailed Student’s t-test. *p < 0 05 vs. EV. [file Image_3.tif]

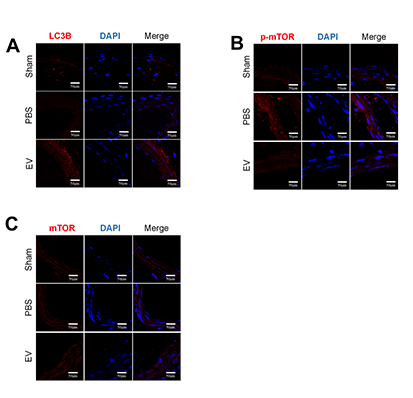

Supplement: Supplementary Figure 4 — The hiPSC-EC-EV treatment could upregulate the endothelial autophagy in carotid artery. (A–C) Representative immunofluorescence images for LC3B (A), p-mTOR (B), and mTOR (C) in the carotid arteries 21 days after the surgery. [file Image_4.tif]
